# Antioxidant Defense Capacity Is Reduced in Thyroid Stem/Precursor Cells Compared to Differentiated Thyrocytes

**DOI:** 10.3390/ijms241411509

**Published:** 2023-07-15

**Authors:** Fiorenza Gianì, Fabio Allia, Maria Antonietta Trovato, Roberta Masto, Gabriella Pellegriti, Riccardo Vigneri

**Affiliations:** 1 Endocrinology, Department of Clinical and Experimental Medicine, University of Catania, Garibaldi-Nesima Medical Center, 95122 Catania, Italy; fiorenza.giani@gmail.com (F.G.); allia.fabio@gmail.com (F.A.); robertamasto88@gmail.com (R.M.); gabriella.pellegriti@unict.it (G.P.); 2 Surgical Oncology, Garibaldi-Nesima Medical Center, 95122 Catania, Italy; maria.trovato@ao-garibaldi.ct.it; 3 Oncology, Department of Clinical and Experimental Medicine, University of Catania, 95123 Catania, Italy

**Keywords:** thyroid stem cells, mature thyrocytes, NRF2-regulated genes, oxidative stress, antioxidant system

## Abstract

There is much evidence linking oxidative stress to thyroid cancer, and stem cells are thought to play a key role in the tumor-initiating mechanism. Their vulnerability to oxidative stress is unexplored. This study aimed to comparatively evaluate the antioxidant capacity of stem/precursor thyroid cells and mature thyrocytes. Human stem/precursor cells and mature thyrocytes were exposed to increasing concentrations of menadione, an oxidative-stress-producing agent, and reactive oxygen species (ROS) production and cell viability were measured. The expression of antioxidant and detoxification genes was measured via qPCR as well as the total antioxidant capacity and the content of glutathione. Menadione elevated ROS generation in stem/precursor thyroid cells more than in mature thyrocytes. The ROS increase was inversely correlated (*p* = 0.005) with cell viability, an effect that was partially prevented by the antioxidant curcumin. Most thyroid antioxidant defense genes, notably those encoding for the glutathione-generating system and phase I detoxification enzymes, were significantly less expressed in stem/precursor thyroid cells. As a result, the glutathione level and the total antioxidant capacity in stem/precursor thyroid cells were significantly decreased. This reduced antioxidant defense may have clinical implications, making stem/precursor thyroid cells critical targets for environmental conditions that are not detrimental for differentiated thyrocytes.

## 1. Introduction

Oxidative stress is a condition characterized by an excess of highly reactive molecules, the most important class being reactive oxygen radicals (ROS). Both endogenous (e.g., mitochondria and NADPH oxidase) and exogenous (e.g., environmental pollutants and endocrine disruptors) sources can generate an excess of free radicals, which may influence intracellular signaling and, when exceeding the cell antioxidant capacity, accumulate and cause oxidative damage to cell structures such as DNA, lipids, and proteins. Oxidative stress may therefore have harmful effects on the biology of the cell, leading to functional abnormalities and malignant transformation [1,2,3,4]. 

Thyroid cells have a peculiar relationship with oxidative stress because iodide metabolism and thyroid hormone synthesis require the continuous generation of hydrogen peroxide (H_2_O_2_), a highly reactive oxygen species [5,6,7]. These cells therefore need a complex antioxidant system against endogenous and exogenous oxidative stress.

The transcription factor NRF2 (nuclear factor erythroid 2-related factor 2) has recently been recognized as the master regulator of the thyroid cell antioxidant response [8,9,10,11,12]. It controls the basal and inducible expression of a series of target genes that ensure a quick cellular response to oxidative stress. When ROS excess or other stressors are present, NRF2 dissociates from its repressor KEAP1 (Kelch-like ECH-associated protein 1) and transfers from the cytosol into the nucleus, where it binds to the Antioxidant Responsive Elements (AREs) and induces the transcription of a battery of antioxidant genes, including oxide-reductase enzymes such as superoxide dismutase (SOD) and catalase (CAT), as well as genes involved in the thioredoxin (TRX) and the glutathione (GSH) systems [13,14,15,16] and also components of the detoxifying system [4,17,18]. When this complex defensive system is impaired or when external oxidants and xenobiotics increase free radical accumulation, adverse consequences may follow, including functional abnormalities, genomic instability, and malignant transformation [19,20,21,22].

The peculiar oxidative condition of thyroid cells is a potential reason for the high frequency of benign and malignant tumors in this gland [23], which has a mutation rate much higher than other tissues [24], although it has a very low proliferation rate [25].

Oxidative stress is a risk factor for thyroid cancer [20,26,27], and it correlates with more aggressive features because ROS may repress genes involved in thyroid differentiation [28]. The antioxidant protection system of undifferentiated thyroid stem cells may therefore be important to preserve their normal biology, regulating the balance between stemness maintenance and the activation of self-renewal and differentiation [29,30,31,32]. However, the antioxidant capacity of thyroid stem/precursor cells has never been explored.

In this study, we evaluated the biological response of primary human thyroid cell cultures to oxidative stress induced by menadione, a well-characterized compound known to generate ROS-based cell stress [33,34,35]. Two different three-dimensional thyroid cell spheroids at a different state of differentiation were studied: stem/precursor thyroid cells and mature thyrocytes. Herein, we found that immature thyroid cells are more susceptible to menadione damage. This menadione effect was partially mitigated by curcumin, a natural phenolic compound that is known to activate the NRF2 pathway and provide antioxidant stress protection [36,37,38]. We also compared the expression of a set of representative genes involved in the thyroid antioxidant defense system, the total antioxidant capacity, and the GSH content in stem/precursor thyroid cells and in mature thyrocytes. The results indicate that immature thyroid cells have a reduced expression of antioxidant genes and decreased content of antioxidant factors and are, therefore, more vulnerable to oxidative stress.

## 2. Results

### 2.1. Differences in Morphology and Gene Markers in the Two Cell Models Used

**Figure 1:**
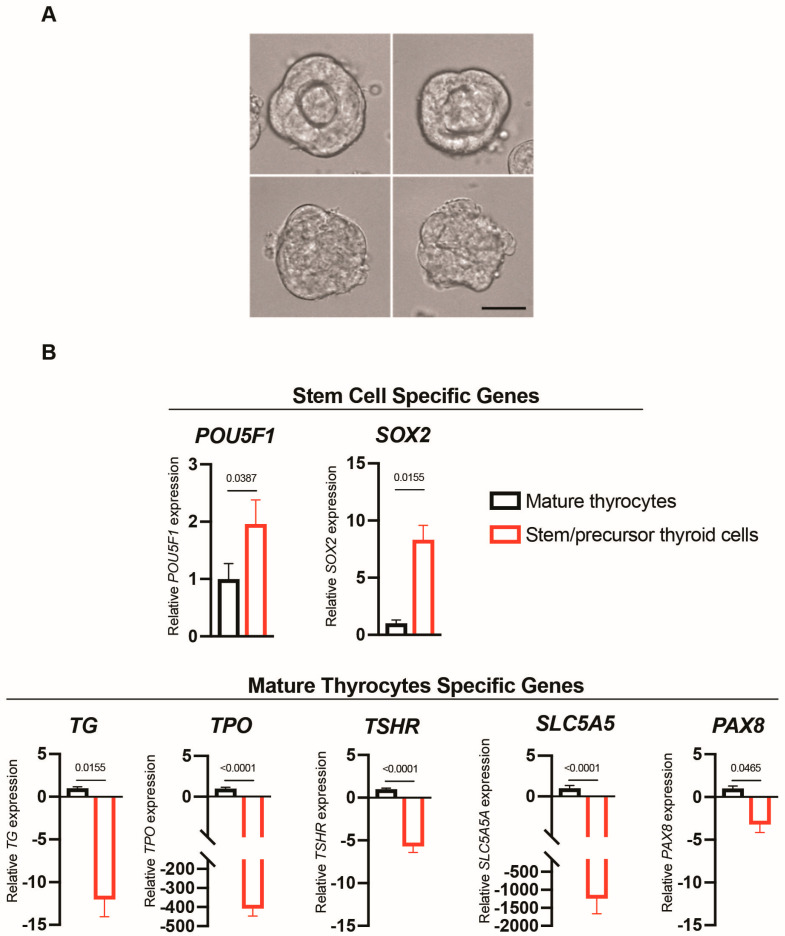
Characteristics of mature thyroid cell spheroids and stem/precursor thyroid cell spheroids. (**A**) Representative phase-contrast microscopy images of mature thyroid cell spheroids (top) and stem/precursor thyroid cell spheroids (bottom) collected 10 days after seeding. Magnification 40× (scale bar 30 µM). (**B**) Expression of stemness genes and thyroid-specific genes in spheroids of differentiated thyrocytes (black bars) and in spheroids of stem/precursor thyroid cells (red bars). Mean values ± S.E.M. of independent experiments using cells from the thyroid of four different patients are shown. Significance (*p*-value) of the difference between mature thyrocytes vs. stem/precursor thyroid cells is indicated for each gene.

Stem/precursor thyroid cell spheroids and mature thyroid cell spheroids were first examined to demonstrate the diverse differentiation level of the two models obtained from the same human thyroid tissue. Spheroids from both mature and immature thyroid cells have a similar size and shape but in mature thyroid cell spheroids thyrocytes appear to remodel into a follicle-like structure with internal lumen-like cavities (Figure 1A). 

Thyroid-specific genes (thyroglobulin, thyroid peroxidase, thyroid stimulating hormone receptor, solute carrier family 5 member 5, and paired box 8) were expressed at a much higher level in mature thyrocyte spheroids, while stemness gene expression (POU class 5 homeobox and SRY box transcription factor 2) was significantly higher in stem/precursor thyroid cell spheroids, demonstrating the diverse differentiation level of the two thyroid cell models prepared from the same thyroid tissues (Figure 1B).

### 2.2. Menadione Effect on ROS Generation 

**Figure 2:**
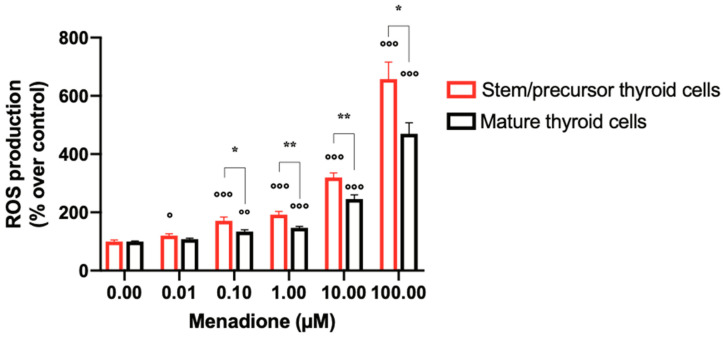
Menadione induces higher levels of ROS generation in stem/precursor thyroid cells compared to mature thyrocytes. Stem/precursor thyroid cells and mature thyrocytes were exposed to increasing concentrations of menadione for 30 min, then stained with 5 µM of CellROX Green Reagent by adding the probe to the medium and incubating the cells at 37 °C for 30 min. The fluorescence intensity was measured using the multiplate reader Victor3 (PerkinElmer) at 485 nm excitation and 535 nm emission wavelengths. ROS generation was expressed as percent changes over untreated cells. Histograms represent the mean values ± S.E.M of three independent experiments using thyroid cells from three different subjects. ° *p* < 0.05; °° *p* < 0.01; and °°° *p* < 0.001 comparing ROS production in the presence of menadione to basal values (untreated cells). * *p* < 0.05 and ** *p* < 0.01 comparing ROS production by stem/precursor thyroid cells in the presence of menadione to ROS levels measured under the same condition in mature thyrocytes.

Under basal conditions, no significant difference in intracellular ROS levels was observed between spheroids from either mature or immature thyroid cells. Exposure to menadione for 30 min led to a clear increase in ROS in thyroid cells. The increase, however, was significantly greater (*p* = 0.0066 by two-way ANOVA) in stem/precursor thyroid cells: at all concentrations tested, the menadione effect was more pronounced in stem/precursor thyroid cells than in differentiated thyrocytes (*p* < 0.05 via Student *t*-test) (Figure 2). Moreover, at the lowest concentration (0.01 µM of menadione), the increase in comparison to control cells was only significant in stem/precursor thyrocytes. The difference in ROS generation (ratio between the ROS increase in the two cell types) decreased progressively at higher menadione concentrations (Figure 2). These data indicate that stem/precursor thyroid cells are more sensitive than mature thyrocytes to the effect of agents causing oxidative stress and that this effect also occurs at concentrations that do not affect differentiated thyrocytes.

### 2.3. Menadione Effect on Thyroid Cell Viability

**Figure 3:**
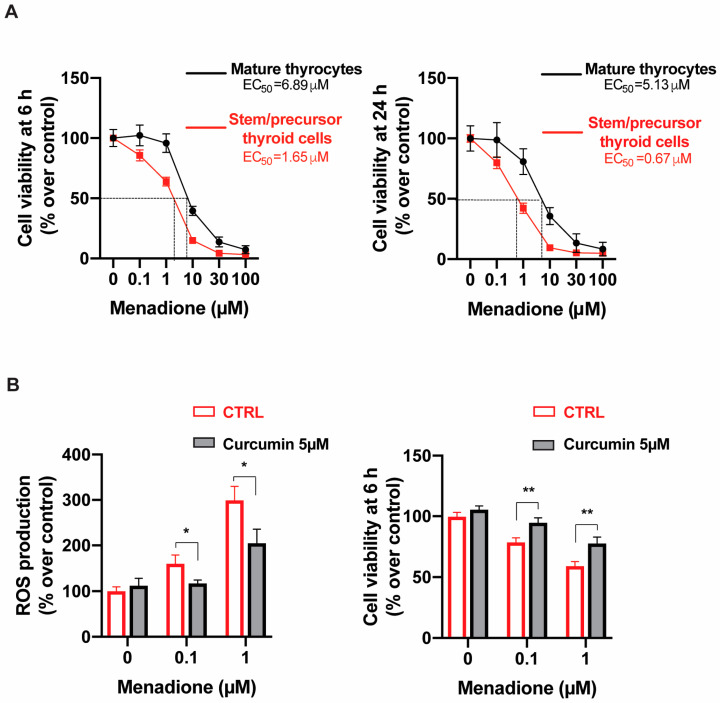
Stem/precursor thyroid cells are more susceptible to menadione toxicity than mature thyrocytes. (**A**) Dose–response curve of immature and mature thyrocytes exposed for 6 (left panel) and 24 h (right panel) to increasing concentration of menadione. Cell viability was determined via RealTime-Glo MT Cell Viability Assay (Promega, Madison, WI, USA). Results are expressed as percent of untreated cells and reported as mean ± S.E.M of three independent experiments using thyroid cells from three different subjects. EC_50_ values were calculated using Graphpad Prism 8 and statistical significance was calculated using two-way ANOVA. (**B**) Curcumin pretreatment attenuates menadione-induced ROS generation and improves stem/precursor thyroid cell viability. Immature thyroid cells were preincubated for 18 h with 5 μM of curcumin, followed by treatment with 0.1 or 1 μM of menadione for an additional 30 min to evaluate ROS generation (left panel) or for 6 h for the cell viability assay (right panel). * *p* < 0.05 and ** *p* < 0.01 compared to the presence or absence of curcumin. CTRL = untreated cells used as control.

To evaluate the biological consequences of the increased susceptibility to oxidative stress, we then measured the effect of menadione on cell viability. Incubation with increasing concentrations of menadione for 6 and 24 h reduced viability in a dose- and time-dependent manner, and this effect was significantly greater in stem/precursor thyroid cells (Figure 3A). At 6 h, the calculated EC_50_ for menadione was 1.65 μM of menadione in immature vs. 6.89 μM in mature thyrocytes (*p* < 0.001). This difference increased from 4- to nearly 8-fold at 24 h (*p* < 0.001) (Figure 3A), indicating that stem/precursor thyroid cells have reduced resistance to oxidative stress caused by menadione and that the difference with mature thyrocytes increases with more chronic exposure. Dose-dependent cell viability was inversely proportional to the amount of ROS generated via exposure to menadione (*p* = 0.005).

### 2.4. The Antioxidant Curcumin Reduces Menadione-Induced ROS Production and Menadione Toxicity in Stem/Precursor Thyroid Cells

When stem/precursor thyroid cells were treated with the antioxidant curcumin (5 μM for 18 h), a significant increase in the expression of the *NQO1, SLC7A11, EPHX1,* and *TXNRD1* genes was observed (Supplementary Figure S1). At the same time, in curcumin-treated cells, exposure to menadione caused reduced ROS production and ameliorated cell viability. In fact, the presence of curcumin significantly (*p* < 0.01) reduced the toxic effect of low doses (0.1 and 1.0 μM) of menadione on immature thyroid cell viability (Figure 3B). 

### 2.5. Antioxidant Gene Expression Is Reduced in Stem/Precursor Thyroid Cells Relative to Differentiated Thyrocytes

To better understand the cause of the reduced antioxidant defense capacity of thyroid stem/precursor cells, we comparatively measured the gene expression of a large panel of components of the thyroid antioxidant and detoxification systems in both immature and differentiated thyrocytes. To this end, we categorized the thyroid antioxidant components according to their preeminent function (Table 1), considering that in the thyroid, antioxidant defense is a very complex process that includes multiple interactions between the antioxidant systems and thyroid-specific biology. 

#### 2.5.1. Master Regulators of the Antioxidant Defense

The expression of both *NFE2L2*, coding for NRF2, the master transcriptional regulator of thyroid antioxidant and cytoprotective pathways, and *KEAP1*, coding for the redox-sensitive NRF2 inhibitor KEAP1, was significantly (*p* < 0.0159 and <0.0289, respectively) reduced in immature thyroid cells to approximately 70% of the values in mature thyrocytes (Table 1). 

#### 2.5.2. Hydrogen Peroxide Producing Enzymes

Thyroid hormone synthesis requires appropriate amounts of H_2_O_2_ generated by members of the NADPH oxidase (NOX) family [5,7]. Among them, a preeminent role is played by dual oxidases DUOX2 and DUOX1. As expected, both genes coding for DUOX1/2 were reduced in immature thyroid cells but the decrease was only statistically significant for DUOX2 (Table 1). In contrast, *NOX4*, another NOX family member that produces both H_2_O_2_ and superoxide anions (O_2_^−^) in a continuous manner [39,40], was significantly increased in stem/precursor thyroid cells (Table 1).

#### 2.5.3. Antioxidant Enzymes and Glutathione 

Differentiated follicular thyroid cells are protected from O_2_^−^ toxic effects by superoxide dismutases (SODs), three isoenzymes that convert O_2_^−^ to H_2_O_2_ in different cellular compartments [41,42]. All *SOD* expression levels were significantly decreased in stem/precursor thyroid cells (Table 1). 

**Figure 4:**
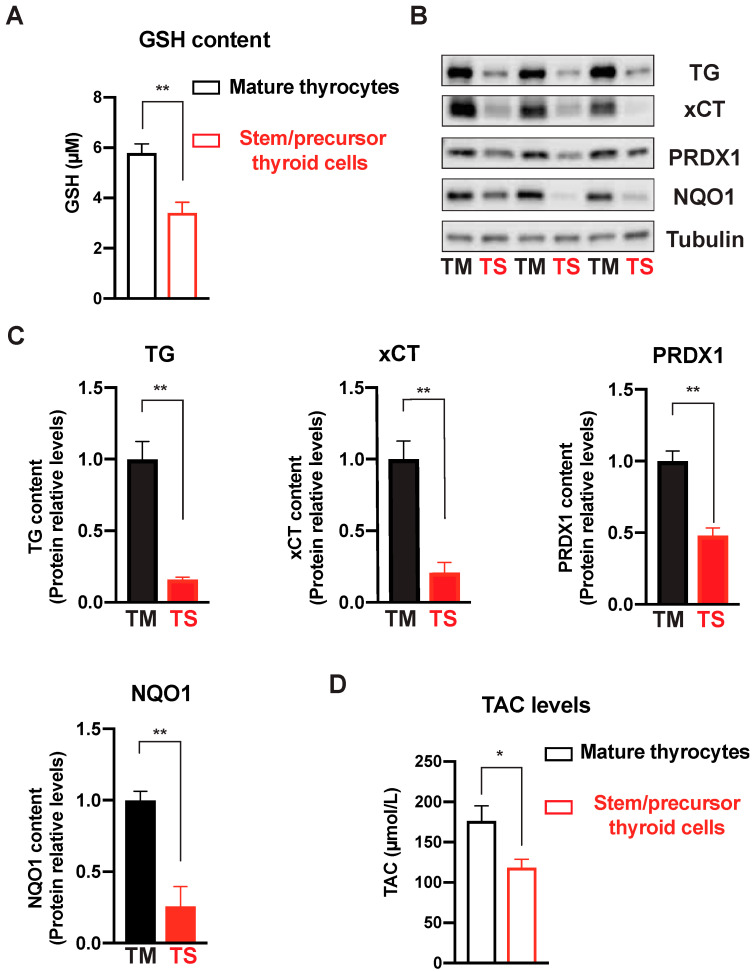
Stem/precursor thyroid cells have reduced antioxidant factor content. (**A**) The intracellular content of glutathione (GSH) in lysate from human immature and mature thyrocytes was measured using the GSH-Glo assay kit (Promega). Luminescence was detected with a Victor3 microplate reader. GSH concentrations were calculated via interpolation from the GSH standard curve. Histograms indicate the mean value ± S.E.M of separate experiments carried out using thyroid cells from four different subjects. ** *p* < 0.01. (**B**) Protein levels of TG (thyroglobulin, a thyroid follicular cell differentiation marker), xCT (a cysteine/glutamate antiporter crucial for providing substrates for glutathione synthesis), PRDX1 (a pivotal component of the thioredoxin–peroxiredoxin system), and NQO1 (a quinone dehydrogenase important for the phase II detoxication system) were measured in immature (TS) and mature thyroid cells (TM) from three different patients via Western immunoblotting. (**C**) Histograms represent the mean ± S.E.M. of densitometric readings normalized to tubulin and expressed as percent of values in mature thyrocytes. ** *p* < 0.01. (**D**) Total Antioxidant Capacity (TAC) in culture medium from thryospheres and mature thyrocytes was measured using the ZellX TAC assay. TAC levels were calculated via interpolation with a ferrous chloride standard curve. Histograms indicate the mean value ± S.E.M from four different subjects. * *p* < 0.05.

As previously mentioned, H_2_O_2_ is a vital component for thyroid function, but any H_2_O_2_ excess must be promptly degraded to prevent oxidative damage. A variety of systems, including the thioredoxin–peroxiredoxin system, glutathione peroxidase, and catalase, carry out this task [13,43]. Genes encoding these factors were reduced in immature thyroid cells (Table 1). In particular, we found a significant decrease in the expression of the thioredoxin–peroxiredoxin-system-related genes *PRDX1* and *TXNRD1* and an even greater decrease in the expression of genes involved in the glutathione antioxidant system. *GPX3* (glutathione peroxidase) and *SLC7A11* (solute carrier family 7 member 11 that encodes xCT, a cysteine/glutamate antiporter crucial for providing substrates for glutathione synthesis) were both significantly reduced to approximately less than 15% compared to differentiated thyrocytes. In addition, the expression of *GSR*, encoding an enzyme that reduces oxidized glutathione disulfide (GSSG) to GSH, was significantly reduced, although to a lesser degree (Table 1). The decreased expression of the genes of the glutathione antioxidant system was confirmed by the significant reduction in stem/precursor thyroid cells (3.44 ± 0.41 vs. 5.92 ± 0.40 μM in differentiated thyrocytes, *p* < 0.01) of intracellular GSH, the most abundant cell antioxidant that plays a major role as a free radical scavenger and detoxifying agent (Figure 4A).

#### 2.5.4. NADPH-Generating Enzymes 

Many of the antioxidant enzymes indicated above require NADPH as a reducing agent. In stem/precursor thyroid cells, the expression of the genes encoding the enzymes involved in the constant generation of NADPH was also markedly reduced (approximately 20–30% of values observed in differentiated thyrocytes) (Table 1). 

#### 2.5.5. Detoxification Systems

Thyroid cells, like all living cells, have multiple mechanisms for the removal of endogenous toxins and environmental toxicants. Additionally, genes encoding the enzymes of the detoxification system were significantly less expressed in immature thyroid cells (Table 1). This decrease was particularly relevant for phase I detoxification enzymes, which oxidize, reduce, and hydrolyze harmful compounds, making them more soluble and favoring their excretion via conjugation (phase II) and transport (phase III) systems. The genes encoding AKR1C1 (acting mainly as NADPH-dependent ketosteroid reductase) [44] and AOX1 (an oxidase with broad substrate specificity that is regulated by the NRF2 pathway) [18] were expressed at less than 10% in stem/precursor thyroid cells compared to differentiated thyrocytes. Additionally, *NQO1*, encoding NAD(P)H:quinone oxidoreductase 1, a protective factor with the ability to catalyze the detoxification of quinones and thus prevent reactive semiquinone and ROS, was downregulated in immature compared to mature thyrocytes.

### 2.6. Antioxidant Enzymatic Protein Measurements and Total Antioxidant Capacity 

Western blot analysis further indicated a significant decrease in some representative proteins of the antioxidant system in stem/precursor thyroid cells, validating the reduced gene expression that we had observed. Compared to those in mature thyrocytes, the protein levels of xCT (*p* = 0.005), PRDX1 (*p* = 0.004), and NQO1 (*p* = 0.008) were all significantly reduced in immature thyroid cells. In the same experiment, the distinct thyroid differentiation level of the two thyroid cell models was confirmed by the different TG contents (*p* = 0.003) (Figure 4B,C).

Finally, the total antioxidant capacity (TAC) measured in stem/precursor thyroid cells (118.2 ± 10.6 µmol/L) was markedly lower than that found in the differentiated thyrocytes of the same individuals (175.9 ± 18.9 µmol/L, *p* = 0.047) (Figure 4D).

## 3. Discussion

Our study demonstrates that thyroid-derived human stem/progenitor cells have reduced resiliency against oxidative stress in comparison with differentiated thyroid cells. When exposed to the redox cycling agent menadione, immature thyrocytes accumulate a greater amount of ROS and have significantly reduced viability. The relationship between the increase in ROS and reduced cell viability is supported by the significant inverse correlation between the two biological effects and by the observation that when ROS production is reduced by the presence of the antioxidant curcumin, the toxic effect of menadione on thyroid stem/precursor cell viability is also reduced.

Menadione toxicity is dose- and time-dependent for both mature and immature thyroid cells, but this effect is significantly greater in immature thyrocytes at all concentrations tested. Moreover, at a very low concentration (10 nM), menadione increased ROS production in stem/precursor thyroid cells but not in mature thyrocytes.

In differentiated thyroid follicular cells, redox homeostasis is ensured by a variety of antioxidant systems that encompass enzymatic and nonenzymatic agents that protect cells from both endogenously and exogenously generated ROS. This complex redox regulation is mainly aimed at protecting thyroid cells from the endogenous oxidative stress produced by the continuous H_2_O_2_ generation necessary for thyroid hormone synthesis. Excess ROS not utilized for thyroid hormone production (or originated by external stressors) is neutralized and eliminated by the internal antioxidant system. In addition to its preeminent role in counteracting the permanent, high-level production of oxidative radicals, components of the thyroid antioxidant system are also connected with other mechanisms of thyroid function; for instance, the transcription factor NRF2, the major regulator of the thyroid antioxidant system, also exerts a pleiotropic role in modulating thyroglobulin synthesis and iodination [8,9].

Stem/precursor thyroid cells have quite a different biology compared to mature thyrocytes and express most of the antioxidant factors at a significantly lower level. Immature thyroid cells do not uptake iodine and do not synthesize thyroid hormones: these functions are not present or are present at a very low level in these cells and, therefore, they are exposed to a lower level of endogenously generated oxidative stress and require less antioxidant defense. Along with their lower expression of ROS-generating enzymes (such as DUOX), stem/precursor thyroid cells also have lower expression levels of the specific antioxidant enzymes involved in H_2_O_2_ elimination such as catalase, SODs, PRDX1, and TXNRD1 (Table 1). However, stem/precursor thyroid cells, like other cell types, can be exposed to oxidative stress induced by environmental toxic agents. External sources of ROS, such as phthalates, bisphenols, pesticides, heavy metals, and other anthropogenic chemicals, frequently occur in the industrialized environment and may cause oxidative stress in these cells with adverse consequences that include abnormal function and malignant transformation [19,20,21,22]. This risk is especially concerning in pregnant women in terms of the thyroid of the developing fetus and in early infancy [45], when stem cells are more represented and active. 

In addition, immature cells require an efficient antioxidant and detoxification system to quench excessive ROS produced by external stressors and to eliminate xenobiotics. The response to menadione shows that in immature thyroid cells, the overall capacity to respond to oxidative stress is reduced and that toxicants may adversely affect their biology even at concentrations that are not detrimental for mature thyroid cells. The greatly reduced expression of genes of the glutathione system and of phase-I detoxifying enzymes (7–16 times less than in mature thyrocytes; Table 1) appears to be the most important factor leading to the deficient antioxidant/detoxifying capacity of stem/precursor thyroid cells. Moreover, the decreased expression (3–5 times less than mature thyrocytes) of NADPH-generating enzymes, the major electron donor for the regeneration of reduced GSH and thioredoxins, probably helps further impede the antioxidant function in these immature cells. 

Increasing evidence indicates that the redox status is a regulator of stem cell function, influencing the balance between self-renewal and differentiation processes [29,30,46,47]. The reduced antioxidant and detoxifying capacity of immature thyroid cells may have important clinical implications, since these cells may become critical targets for environmental toxicants [48,49] even at very low concentrations. Distorted differentiation may be a pathogenetic mechanism for the susceptibility of derived thyrocytes to thyroid diseases and thyroid cancer [50].

One limitation of our study is that the antioxidant defense capacity of thyroid cells was tested using only menadione, an oxidative chemical used in a broad spectrum of studies. In the real world, however, oxidative stress can be produced by different chemicals in different mixtures, and these may elicit different types of antioxidant responses [51].

Another limitation is that we investigated the molecular biology of immature thyroid cells using a well-accepted thyroid cell model but characterized by a heterogeneous population of stem/precursor thyroid cells at different stages of differentiation, each with their own pattern of gene expression [52]. It is reasonable to expect that antioxidant gene expression will change with increasing differentiation and that stem cells could be the most fragile component, possibly damaged by stressors that are less detrimental for thyrocytes at a more advanced stage of differentiation. 

In conclusion, our study provides the first characterization of the reduced antioxidant defense capacity of human thyroid stem/precursor cells, which draws attention to the possible biological damage (including altered function and propensity to carcinogenesis) that environmental pollutants, even at levels that are not harmful for mature thyrocytes, may cause to the thyroid.

## 4. Materials and Methods

### 4.1. Human Thyroid Cells

Normal thyroid tissues (*n* = 22) were obtained from euthyroid female patients who provided written informed consent before undergoing thyroid surgery for either benign or malignant thyroid nodules. The study was approved by the Institutional Ethical Committee (n.12/2015/CECT2) and completed in accordance with the declaration of Helsinki.

Primary thyroid cell cultures were established from histopathological normal thyroid tissue as previously reported [48,53]. Briefly, human thyroid tissue specimens were minced and digested in collagenase IV (1 mg/mL; Sigma-Aldrich, St. Louis, MO, USA). The obtained cell suspension was then collected via centrifugation at 400× *g*, washed twice and subsequently cultured in RPMI 1640 culture medium (Sigma-Aldrich), 2 mM glutamine (Sigma), 2.5% heat-inactivated fetal bovine serum (FBS, Invitrogen, Waltham, MA, USA), B-27 (1:100, Gibco), insulin-transferrin-sodium selenite liquid medium supplement (ITS, 1:200, Gibco), and epidermal growth factor (EGF, 1 ng/mL; Sigma-Aldrich). 

These primary culture thyrocytes were used to obtain two different three-dimensional thyroid cell spheroids with a different level of differentiation. First, the thyrocytes were trypsinized into single cells and seeded at a density of 6.0 × 10^5^ cells per well in AggreWell^TM^ 400 6-well plates (StemCell Technologies, Vancouver, BC, Canada) according to the manufacturer’s instructions. AggreWell plates provide a method to produce a large number of separate 3D spheroids since each well contains a standardized array of microwells (approximately 5900 per well, with each being 400 µm in size) where single spheroids grow uniform in size and shape [54]. Following this procedure:

(a) To obtain immature (stem/precursor) thyroid cell spheroids, cells were cultured in serum-free RPMI medium without phenol-red and supplemented with 20 ng/mL EGF and B27 without five antioxidants (1:100, Gibco) for antioxidant-free conditions. 

(b) To generate mature thyroid cell spheroids, thyrocytes were cultured in a similar medium with a lower concentration of EGF (1 ng/mL) and supplemented with 1 mU/mL bovine TSH. Cells were allowed to aggregate in spheroids for 7 days, and the medium was replaced every 2–3 days. 

### 4.2. Cell Exposure to Oxidative Stress

To evaluate the response to oxidative stress, stem/precursor thyroid cells and mature thyrocytes were exposed for the indicated times and concentrations to the free-radical-generating agent menadione (Vitamin K3, Sigma-Aldrich), a synthetic analog of 1,4-naphthoquinone with redox cycling activity used in many studies to induce oxidative stress [33,34]. Stock solutions were freshly prepared in DMSO at 5.8 mM menadione, and working solutions were prepared in RPMI medium. Biological changes due to menadione were also evaluated in the presence of 5 µM of curcumin (Santa Cruz Biotechnology, Dallas, TX, USA), a phytochemical with protective activity against oxidative stress [36,37].

### 4.3. ROS Measurement

ROS were assessed using the cell-permeable CellROX Green reagent (Thermo Fischer, Waltham, MA, USA), a fluorogenic probe for measuring oxidative stress in living cells that exhibits bright-green photostable fluorescence upon oxidation by ROS.

Mature thyrocytes and precursor/stem cells disaggregated from spheroids were seeded at a cell density of 10^4^ cells/well in black 96-well plates in RPMI medium without phenol-red supplemented with 2% FBS. After 24 h, the cells were cultured overnight in RPMI starvation medium, and then, menadione was added at the indicated concentrations for 30 min. Five µM of CellROX^®^ Green was then added, and the cells were incubated for an additional 30 min. Fluorescence was measured using a multiplate reader.

### 4.4. Cell Viability 

Immature and mature thyroid cell spheroids were collected and seeded into white 96-well plates (PerkinElmer, Waltham, MA, USA) treated with antiadherence rinsing solution (StemCell Technologies) and incubated overnight. Then, the spheroids were incubated with RealTime-GloTM Assay Reagent (Promega) and exposed to increasing concentrations of menadione. Luminescence was measured at the indicated times using a Victor3 microplate reader. EC_50_ values (50% decrease in viable cells) were calculated using Prism 8.0 software.

### 4.5. Gene Expression

Total RNA was isolated using the RNeasy Mini Kit (Qiagen, Hilden, Germany), according to the manufacturer’s instructions. gDNA removal and cDNA synthesis were performed using the QuantiTect Rev Transcription Kit (Qiagen), and qPCR was performed in a 7500 Real-Time PCR System (ABI). Primer sequences for qPCR are shown in Table 2. Data were normalized to the stably expressed reference genes RPS3 and RPS6. Data were analyzed using the 2^−∆∆CT^ method and were presented as fold regulation compared to mature thyroid cells.

Briefly, fold-change (2^−∆∆CT^) values were obtained by dividing the normalized gene expression (2^−∆CT^) in the mature thyrocyte samples by the normalized gene expression (2^−∆CT^) in the stem/precursor thyroid cell samples. Values greater than one indicate positive or up-regulation, and the fold-regulation equals the fold-change. Values less than one indicate negative or down-regulation, and the fold-regulation is the negative inverse of the fold-change. The *p* values were calculated using Student’s *t*-test of the replicate (2^−∆CT^) values for each gene in the mature thyrocyte and stem/precursor thyroid cell groups.

### 4.6. Western Immunoblotting 

Western blots were performed as previously described [48,53,55] in lysed mature and immature thyrocyte spheroids. The following primary antibodies were used at 1:1000 dilution: Anti-SLC7A11 (D2M7A) and antitubulin (D3U1W) (both from Cell Signaling Technology) and anti-PRDX1 (D5G12), antithyroglobulin (D-9), and anti-NQO1 (A180) (all from Santa Cruz). 

### 4.7. Glutathione Measurement

Mature and immature thyroid cells were seeded at a density of 10^4^ cells/well into white 96-well clear-bottom plates. GSH was determined using the GSH-GLO Glutathione assay (Promega) according to the manufacturer’s protocol. Luminescence was detected with a Victor3 microplate reader. GSH concentrations were calculated from the standard curve after background subtraction, and the values were normalized to the total cell content determined via crystal violet staining.

### 4.8. Total Antioxidant Capacity (TAC)

TAC was measured in the culture medium of both mature and immature thyroid cell spheroids using the ZellX TAC assay kit based on the antioxidant power of the biological sample to reduce ferric ions (Fe^3+^) to ferrous ions (Fe^2+^) in the FRAP Color solution under acidic conditions. The generated blue-color product, proportional to total antioxidant status, was read at 560 nm. 

### 4.9. Statistical Analysis

Statistical analyses were performed with Prism 8.0 statistics software. The results are shown as the mean ± standard error of the mean (S.E.M.) of at least three thyroid cell preparations from different donors and were analyzed via Student’s *t*-test or via two-way ANOVA. Correlation analysis was performed using the Spearman correlation coefficient. Data were assumed to be significantly different when the *p* value was <0.05.

## Figures and Tables

**Table 1:** Expression of selected genes involved in human thyroid cell antioxidant system. Changes in stem/precursor thyroid cells relative to differentiated thyrocytes obtained from the same individual are indicated as Fold Regulation. Values indicate the mean values (± S.E.M.) calculated from four different patients.

*Gene Symbol and Extended Name*		*Fold Regulation (Mean ± S.E.M.)*	*p Value*
**Master Regulators of the Antioxidant Defense**			
***NFE2L2***	Nuclear factor, erythroid-2-like 2	−1.40 ± 0.08	0.0159
***KEAP1***	Kelch-like ECH-associated protein 1	−1.45 ± 0.12	0.0289
**Hydrogen Peroxide Producing Enzymes**			
***DUOX1***	Dual oxidase 1	−1.65 ± 0.31	0.1259
***DUOX2***	Dual oxidase 2	−2.65 ± 0.43	0.0374
***NOX4***	NADPH oxidase 4	2.04 ± 0.11	0.0093
**Antioxidant Enzymes**			
***SOD1***	Superoxide dismutase 1	−1.58 ± 0.11	0.0019
***SOD2***	Superoxide dismutase 2	−2.35 ± 0.26	<0.0001
***SOD3***	Superoxide dismutase 3	−3.50 ± 0.94	0.0127
***CAT***	Catalase	−1.42 ± 0.05	0.0013
***PRDX1***	Peroxiredoxin 1	−2.71 ± 0.35	0.0040
***TXNRD1***	Thioredoxin Reductase 1	−4.31 ± 0.99	0.0099
**Glutathione antioxidant system**			
***GPX3***	Glutathione peroxidase 3	−6.79 ± 0.97	0.0040
***SLC7A11***	Solute carrier family 7 member 11	−7.78 ± 0.97	<0.0001
***GSR***	Glutathione-disulfide reductase	−1.96 ± 0.25	0.0450
**NADPH-generating enzymes**			
***G6PD***	Glucose-6-phosphate dehydrogenase	−3.17 ± 0.48	0.0003
***PGD***	Phosphogluconate dehydrogenase	−3.49 ± 0.76	0.0003
***ME1***	Malic enzyme 1	−5.57 ± 1.21	0.0002
**Detoxification Systems**			
**Phase I**			
***EPHX1***	Epoxide hydrolase 1	−3.75 ± 0.49	<0.0001
***AKR1C1***	Aldo-keto reductase family 1 member C1	−11.91 ± 3.17	0.0003
***AOX1***	Aldehyde oxidase 1	−15.91 ± 3.35	<0.0001
**Phase II**			
***MGST1***	Microsomal glutathione S-transferase 1	−1.51 ± 0.10	0.0014
***NQO1***	NAD(P)H quinone dehydrogenase 1	−2.24 ± 0.27	0.0038
**Phase III**			
***ABCC1***	ATP-binding cassette subfamily C member 1	−1.57 ± 0.24	0.0039

**Table 2:** Nucleotide sequences of primers used for quantitative PCR.

Category	*Gene*	Primer Direction	Primer Sequence	Amplicon
**Master Regulators of the Antioxidant Defense**	*NFE2L2*	For	TCCATTCCTGAGTTACAGTGTC	228
		Rev	CACTGTCAACTGGTTGGGGT	
	*KEAP1*	For	TGCGTCCTGCACAACTGTAT	199
		Rev	CCAGGAACGTGTGACCATCA	
**Hydrogen Peroxide-Producing Enzymes**	*DUOX1*	For	ACGTGCTGGTCGCTGTTATC	204
		Rev	AAGGGAAGCAACAGAGGGTC	
	*DUOX2*	For	TTAGTTCTGAAGAGGAACGGGG	199
		Rev	TCGGCCTGGTTGATGTCCA	
	*NOX4*	For	TCCGGAGCAATAAGCCAGTC	199
		Rev	ACCCCAAATGTTGCTTTGGT	
**Antioxidant Enzymes**	*SOD1*	For	ACAAAGATGGTGTGGCCGAT	162
		Rev	AACGACTTCCAGCGTTTCCT	
	*SOD2*	For	TCCGGTTTTGGGGTATCTGG	155
		Rev	CGGTGACGTTCAGGTTGTTC	
	*SOD3*	For	AGCTGGAAAGGTGCCCGA	149
		Rev	CTTGGCGTACATGTCTCGGAT	
	*CAT*	For	CTGACTACGGGAGCCACATC	192
		Rev	CATCCAGTGATGAGCGGGTT	
	*PRDX1*	For	CAAAGCCACAGCTGTTATGCC	186
		Rev	GAAGCACCAATCACTTGGCAG	
	*TXNRD1*	For	TGGAGTGCGCTGGATTTCTT	187
		Rev	CCTGGTGTCCCTGCTTCAAT	
**Glutathione** **Antioxidant System**	*GPX3*	For	TACGAGTACGGAGCCCTCAC	160
		Rev	GACCGAATGGTGCAAGCTCT	
	*SLC7A11*	For	ACAGGGATTGGCTTCGTCAT	190
		Rev	GGCAGATTGCCAAGATCTCAA	
	*GSR*	For	TGGCACTTGCGTGAATGTTG	225
		Rev	GCATGGCCACGGATGATTTC	
**NADPH-Generating Enzymes**	*G6PD*	For	GGCCGTGTACACCAAGATGA	212
		Rev	GCAGTGGGGTGAAAATACGC	
	*PGD*	For	AAGATGGTGCACAACGGGAT	218
		Rev	TCCCTGATCTTTGGCAGCAG	
	*ME1*	For	ACCCTCACCTCAACAAGGACT	87
		Rev	TGTTGAAGGAAGGTGGCAACA	
**Detoxification System**	*EPHX1*	For	GCTGACCAACGTCATGCTCT	120
		Rev	ACATAGACCTTCATCCGCTCA	
	*AKR1C1*	For	GAAGCTGGCTTCCGCCAT	158
		Rev	ACCAACTCTGGTCGATGGGA	
	*AOX1*	For	AAACGCCTCGAACCCATCAT	222
		Rev	CTTATGATCCCCCGTCAGGC	
	*MGST1*	For	GACCTCACCCAGGTAATGGA	214
		Rev	TGCGTACACGTTCTACTCTGTC	
	*NQO1*	For	GAGCACTGATCGTACTGGCT	185
		Rev	AAAGTTCGCAGGGTCCTTCA	
	*ABCC1*	For	GAGGACACGTCGGAACAAGT	141
		Rev	CGCATCCACCTTGGAACTCT	
**Mature-Thyrocyte-Specific Genes**	*TG*	Hs00174974_m1		
	*TPO*	Hs00174927_m1		
	*TSHR*	Hs01053846_m1		
	*NIS*	Hs00166567_m1		
	*PAX8*	For	GGCCTTTGTGAATGGCAGAC	243
		Rev	TTCTGGCGTTTGTAGTCCCC	
**Stem-Cell-Specific Genes**	*POU5F1*	Hs01654807_s1		
	*SOX2*	Hs04234836_s1		
**Housekeeping Genes**	*RPS3*	For	CCACTAGAGGTCTGTGTGCC	157
		Rev	CCTCGGAGTTTCCCAGACAC	
	*RPS6*	For	TGTTACTCCACGTGTCCTGC	166
		Rev	AAGTCTGCGTCTCTTCGCAA	

## Data Availability

The data presented in this study are available in the article and Supplementary material.

## Supplementary Materials

The supporting information can be downloaded at: https://www.mdpi.com/article/10.3390/ijms241411509/s1.
