# Supplementary material for: Antioxidant Defense Capacity Is Reduced in Thyroid Stem/Precursor Cells Compared to Differentiated Thyrocytes
Source: Int J Mol Sci. 2023 Jul 15;24(14):11509. doi: 10.3390/ijms241411509 (PMC10380350; doi:10.3390/ijms241411509)

Figure S1. Increased expression of the antioxidant genes ***NQO1, SLC7A11, TXNRD1*** and ***EPHX1*** in stem/precursor thyroid cell spheroids treated with 5 µM curcumin for 18h. mRNA levels were measured by qPCR. Bar graph show the relative mRNA level of genes in the curcumin-treated spheroid compared to control spheroid used as calibrator. Data shown are the mean ± S.E.M. of three independent experiments. (*p< 0.05, **p<0.01, ***p< 0.001).

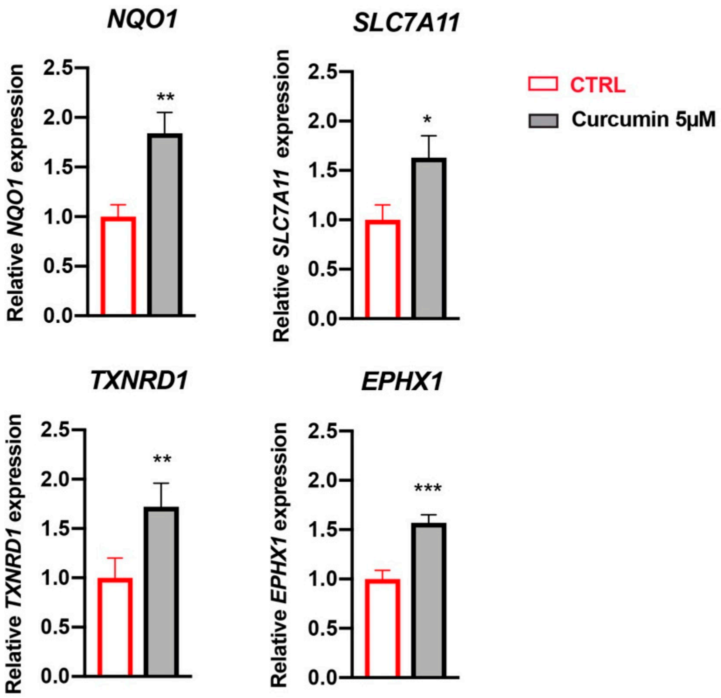

Supplement: Supplementary file 1 [file ijms-24-11509-s001.zip › ijms-2409873-supplementary.pdf]
